# A novel multi-parametric analysis of non-invasive methods to assess animal distress during chronic pancreatitis

**DOI:** 10.1038/s41598-019-50682-3

**Published:** 2019-10-01

**Authors:** Ahmed Abdelrahman, Simone Kumstel, Xianbin Zhang, Marie Liebig, Edgar Heinz Uwe Wendt, Johanna Eichberg, Rupert Palme, Thomas Thum, Brigitte Vollmar, Dietmar Zechner

**Affiliations:** 1 Rudolf-Zenker-Institute of Experimental Surgery, Rostock University Medical Center, Rostock, Germany; 2 Unit of Physiology, Pathophysiology and Experimental Endocrinology, Department of Biomedical Sciences, University of Veterinary Medicine Vienna, Vienna, Austria; 3 Institute of Molecular and Translational Therapeutic Strategies, Hannover Medical School, Hannover, Germany

**Keywords:** Animal behaviour, Animal physiology

## Abstract

Ethical responsibility, legal requirements and the need to improve the quality of research create a growing interest in the welfare of laboratory animals. Judging the welfare of animals requires readout parameters, which are valid and sensitive as well as specific to assess distress after different interventions. In the present study, we evaluated the sensitivity and specificity of different non-invasive parameters (body weight change, faecal corticosterone metabolites concentration, burrowing and nesting activity) by receiver operating characteristic curves and judged the merit of a multi-parametric analysis by logistic regression. Chronic pancreatitis as well as laparotomy caused significant changes in all parameters. However, the accuracy of these parameters was different between the two animal models. In both animal models, the multi-parametric analysis relying on all the readout parameters had the highest accuracy when predicting distress. This multi-parametric analysis revealed that C57BL/6 mice during the course of chronic pancreatitis often experienced less distress than mice after laparotomy. Interestingly these data also suggest that distress does not steadily increase during chronic pancreatitis. In conclusion, combining these non-invasive methods for severity assessment represents a reliable approach to evaluate animal distress in models such as chronic pancreatitis.

## Introduction

Laboratory animals represent a valuable tool for mimicking physiological conditions in humans and are, therefore, still widely used for biomedical research in the pursuit of understanding the pathophysiology of a disease and exploring novel treatments^1,2^. On the other hand, improving animal welfare is important in order to alleviate the suffering of animals. Consequently, the 3Rs (replacement, reduction and refinement of animal experiments) have been coined by Russell and Burch^3^ more than 50 years ago and are now the basis of national and international regulations of animal experiments. In accordance with this concept, the European Union Directive 2010/63/EU and many local institutional animal care and use committees in the USA demand to provide adequate information to assess the potential distress and pain that animals might experience during an experiment^4,5^. This represents an essential component of the harm-benefit analysis when evaluating any project that involves animal experiments^4^. Distress is often defined as an aversive state in which the animal is unable to adapt completely to stressors and the resulting stress and shows maladaptive behaviour^6,7^. Indeed, pain can be seen as just one possible cause of distress, thus suggesting that quantifying distress of animals might be of greater importance for animal welfare than focusing just on the quantification of pain^8^. However, it has been suggested that distress is not always a consequence of pain^9^; e.g. it might be possible that short time pain does not cause maladaptive behaviour. In this study, we follow the concept of Koch *et al*. that one should focus on distress (both pain-related and non-pain-related) when evaluating animal welfare^8^.

The European Union Directive 2010/63/EU provides examples of severity grading for some interventions. For instance, the directive suggests that laparotomy causes moderate distress. Thus, such a model was used in this study as a reference model for evaluating the degree of distress experienced by mice during chronic pancreatitis (CP). Importantly, only few publications provide a profound distress analysis of a specific animal model^10–12^, and there is a crucial need for more objective severity grading of procedures^13^. Additionally, in order to judge the severity of animal models, it is important, first, to validate methods used for distress analysis and, second, to combine these methods to pursue a multi-parametric analysis of distress. Finally, the distress caused by each animal model needs to be compared to a reference.

Animal distress associated can be assessed by various distress indicators. For example, burrowing or nesting activity may reflect the well-being of mice^14,15^. In addition, body weight is used as an indicator of well-being, since animals in pain can have reduced food and water intake^16^. Moreover, many research groups have described that the concentration of faecal corticosterone metabolites (FCM)^17–19^ and plasma corticosterone concentration^20^ may indicate animal distress. Thus, many publications describe multiple methods to score distress^14,15,17–19^. However, little is known about which readout parameters are best suited to diagnose distress. For judging the diagnostic ability of tests and readout parameters, receiver operating characteristic (ROC) curves are widely used in clinical^21–23^ and in experimental settings^24^ Moreover, ROC curves have also been widely used to define the accuracy of psychological distress scores for cancer patients^25,26^.

The pathophysiology of CP remains poorly understood and is still inadequately treated in humans^27^. Moreover, CP inflicts abdominal pain on patients, which affects daily activities as the disease progresses^28,29^. In addition to pain, CP can also lead to endocrine and exocrine pancreatic insufficiency with severe complications^29^. These facts altogether create a crucial need for extensive scientific research in the field of CP in order to explore pathophysiological mechanisms and novel therapeutic approaches. Interestingly, while some animal models for CP do not reproduce all histopathological features of this disease and some animal models have a relatively high mortality rate, repetitive i.p cerulein injections very reliably cause CP^30,31^.

In humans, pain of CP is managed primarily by paracetamol, nonsteroidal anti-inflammatory drugs or opioid analgesics such as tramadol^32^. Nevertheless, pain is still often insufficiently controlled^33^. Recently, microRNAs have been implicated in modulating pain perception^34^. For example, microRNA-21 has been described to contribute to neuropathic pain in mice^35,36^. Since neuropathic pain is considered to be an inherent component of pain in CP^37,38^, inhibition of microRNA-21 might support analgesic regimens and mitigate animal distress associated with CP.

It was the aim of this project to evaluate physical, behavioural and physiological animal distress parameters during CP and to compare their ability to predict distress. We also aimed at exploring the benefit of multi-parametric analysis and compared the distress animals experience during CP to distress after laparotomy.

## Results

### Pathological features and distress caused by CP

**Figure 1:**
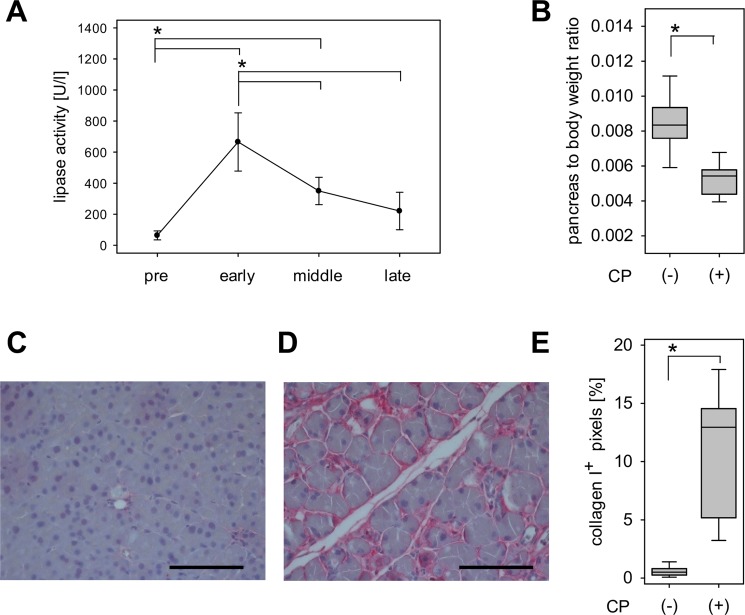
Evaluation of chronic pancreatitis. (**A**) Serum lipase concentration before (pre) and during the early, middle and late phase of cerulein-induced CP; One Way Repeated Measures ANOVA followed by pairwise comparison with Holm-Sidak-Method (*P ≤ 0.041), n = 4. (**B**) Pancreas to body weight ratio of healthy mice (−CP) and mice at the late phases of CP (+CP); Student’s t-test (*P ≤ 0.001), healthy: n = 12, CP: n = 15. Immunohistochemical detection of collagen I accumulation in (**C**) healthy mice and (**D**) mice at the late phase of CP; bar =100 µm. (**E**) Quantification of collagen I deposition in healthy mice (−CP) and at the late phase of CP (+CP); unpaired t-test with Welch’s correction (*P < 0.0001), healthy: n = 8, CP: n = 5.

Plasma lipase activity, indicating damage to the pancreas, was significantly elevated during the early and middle phase of CP (Fig. 1A). However, it was reduced during the middle and late phase when compared to the early phase of CP (Fig. 1A). The pancreas to body weight ratio, as a measurement of tissue atrophy, was significantly reduced in cerulein-injected mice when compared to healthy mice (Fig. 1B). Another feature of CP, fibrosis, was also observed in cerulein-injected animals (Fig. 1C,D). The quantification of collagen I deposition in pancreatic tissue confirmed increased fibrosis in mice with CP (Fig. 1E). Thus, repetitive cerulein injection induced key characteristics of CP in mice.

**Figure 2:**
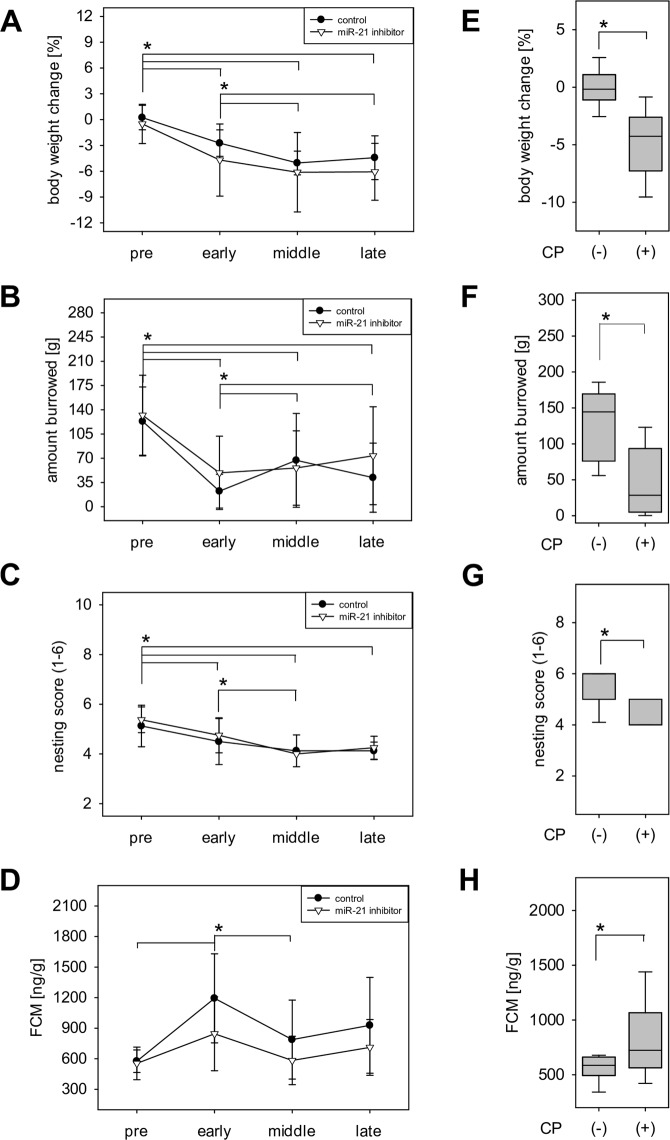
Course of distress during chronic pancreatitis. (**A–D**) Determination of distress parameters in control and microRNA-21 inhibitor treated mice before (pre) and during the early, middle and late phases of CP; control: n = 8, microRNA-21 inhibitor: n = 8. (**A**) Percentage of body weight change; Two Way Repeated Measures ANOVA, followed by pairwise comparison with Holm-Sidak-Method (*P ≤ 0.047). (**B**) Burrowing activity within 2 hours; Two Way Repeated Measures ANOVA, followed by pairwise comparison with Holm-Sidak-Method (*P ≤ 0.002). (**C**) Nesting activity Two Way Repeated Measures ANOVA, followed by pairwise comparison with Holm-Sidak-Method (*P ≤ 0.021). (**D**) Concentration of faecal corticosterone metabolites (FCM); Two Way Repeated Measures ANOVA, followed by pairwise comparison with Holm-Sidak-Method (*P ≤ 0.025). (**E–H**) Comparing distress before (−CP, n = 16) and during the early, middle and late phases of cerulein treatment (+CP, n = 48). (**E**) Percentage of body weight change; Mann-Whitney Rank Sum Test (*P ≤ 0.001). (**F**) Evaluation of burrowing activity; Mann-Whitney Rank Sum Test (*P ≤ 0.001). (**G**) Evaluation of nesting activity; Mann-Whitney Rank Sum Test (*P ≤ 0.001). (**H**) Assessment of faecal corticosterone metabolites concentration; Mann-Whitney Rank Sum Test (*P = 0.016).

We observed a significant reduction of body weight in all phases after induction of CP when compared to the pre-phase, which is the phase before any intervention (Fig. 2A). We also observed a reduction in body weight between the early phase and the subsequent phases, but not between the middle and the late phase (Fig. 2A). Burrowing activity was significantly reduced throughout the course of CP with the deepest reduction in the early phase, thereafter, a slight recovery was observed (Fig. 2B). Nesting activity was significantly reduced from pre-phase to all post induction phases (Fig. 2C). Moreover, the nesting scores were significantly reduced between the early phase and the middle phase, but not between the middle and the late phase. FCM concentration significantly increased in the early phase after induction, followed by a significant decrease in the middle phase (Fig. 2D). We observed no significant differences in any of these readout parameters between animals treated with microRNA-21 inhibitor and animals treated with microRNA-21 control oligonucleotides (Fig. 2A–D). When comparing these distress parameters before and collectively after induction of CP, we noticed a significant reduction in percent body weight change (Fig. 2E), burrowing activity (Fig. 2F) and nesting activity (Fig. 2G) as well as a significant increase in FCM concentration (Fig. 2H). In order to check the validity of the FCM data, we analysed blood corticosterone concentration at different phases of CP. Corticosterone concentration in the blood also significantly increased in the early phase after induction, followed by a significant decrease in the middle phase (Supplementary Fig. S1). However, this hormone remained significantly elevated at all time points after intervention when compared to the pre-phase (Supplementary Fig. S1). There was also a significant increase when collectively comparing blood corticosterone concentration before and after induction of CP (Supplementary Fig. S1). Thus, these data demonstrate that all readout parameters are sensitive enough to score increased distress after induction of CP.

### The accuracy of parameters defining distress after CP

**Figure 3:**
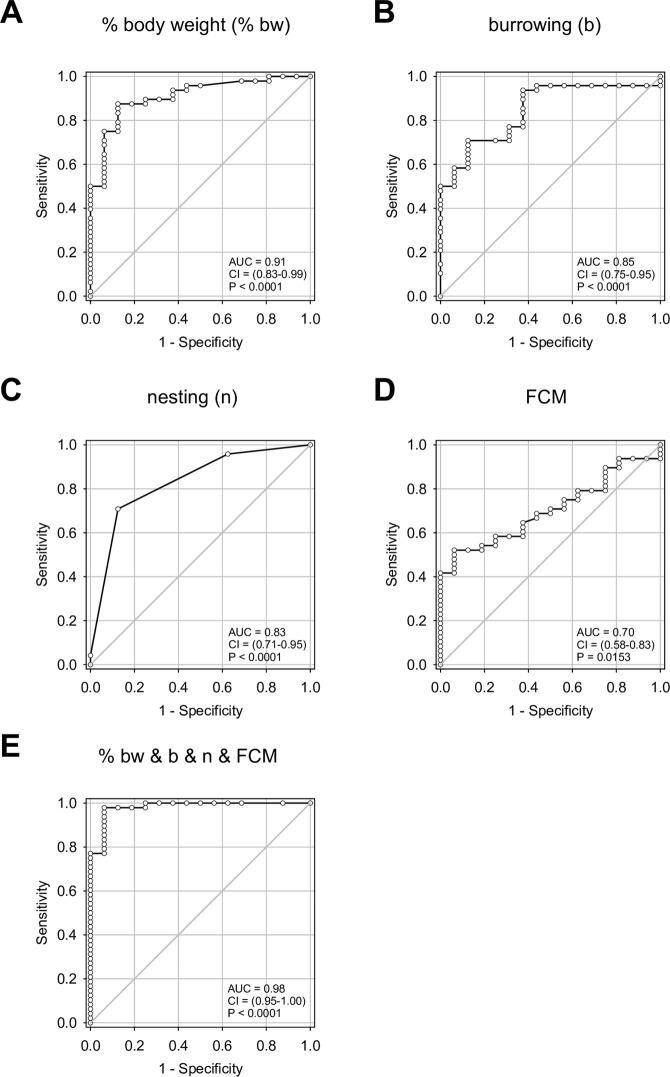
Receiver operating characteristic curve analysis of distress parameters in the chronic pancreatitis animal model. Evaluation of (**A**) percentage of body weight change, (**B**) burrowing activity, (**C**) nesting activity, (**D**) concentration of faecal corticosterone metabolites (FCM) and (**E**) combination of these four parameters after applying binary logistic regression in a multiple model. The area under the curve (AUC) with the respecting confidence interval (CI) and the P value (P) for testing the AUC to be 0.5, are indicated. Before induction of CP: n = 16, after induction of CP: n = 48.

The single readout parameters, percentage of body weight change (Fig. 3A), burrowing activity (Fig. 3B), nesting activity (Fig. 3C) and FCM (Fig. 3D) had discriminatory power to differentiate between animals before and after induction of CP. Moreover, the combination of all four parameters also had discriminatory power to differentiate between animals before and after induction of CP (Fig. 3E). The combination of all four parameters produced the largest area under the curve (AUC = 0.98, 0.95–1.00 confidence interval), suggesting that the combination of all readout parameters allows to differentiate between distress assessed before and after induction of CP with higher accuracy than any of the single readout parameters (Fig. 3A–E). When applying Youden’s index to define the best cut off to differentiate between distress of animals before and after induction of CP, the combination of all readout parameters allows differentiating between animals before and after induction of CP with higher accuracy (96.9%, 89.2–99.6% confidence interval) than any of the single readout parameters (Supplementary Table S1).

### Comparing distress after laparotomy to distress during CP

**Figure 4:**
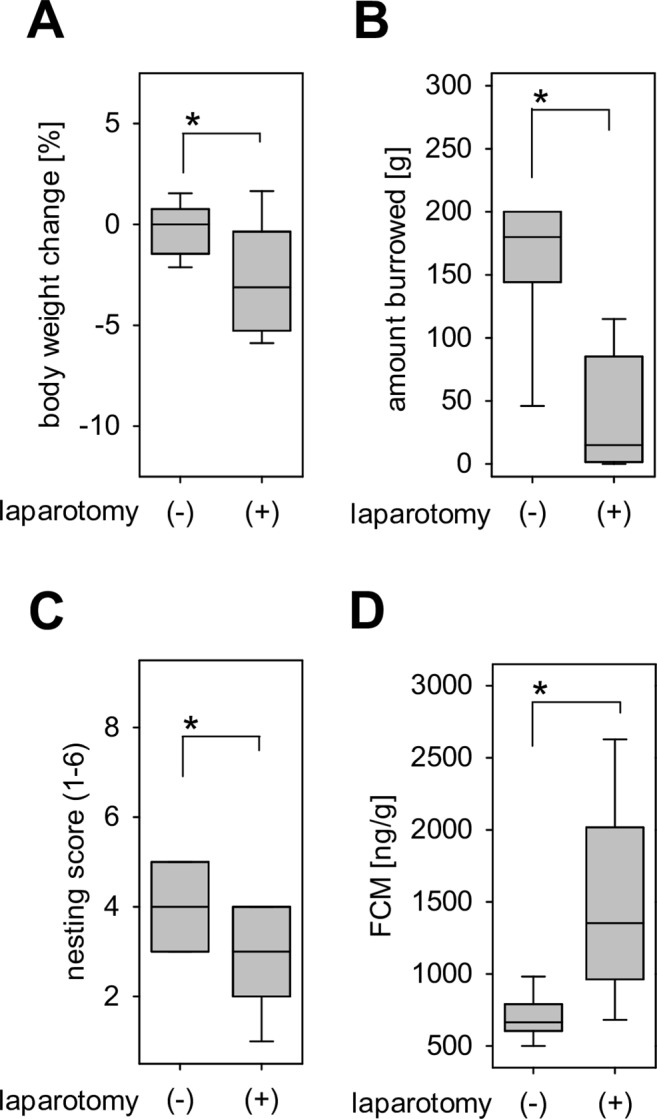
Distress before and after laparotomy. Quantification of distress in mice before (−laparotomy) and after laparotomy (+laparotomy). (**A**) Percentage of body weight change; Paired t-test (*P ≤ 0.001), n = 25. (**B**) Burrowing activity within 2 hours; Wilcoxon signed-rank test (*P ≤ 0.001), n = 25. (**C**) Nesting activity; Wilcoxon signed-rank test (*P = 0.002), n = 25. (**D**) Concentration of faecal corticosterone metabolites (FCM); Paired t-test (*P ≤ 0.001), n = 25.

**Figure 5:**
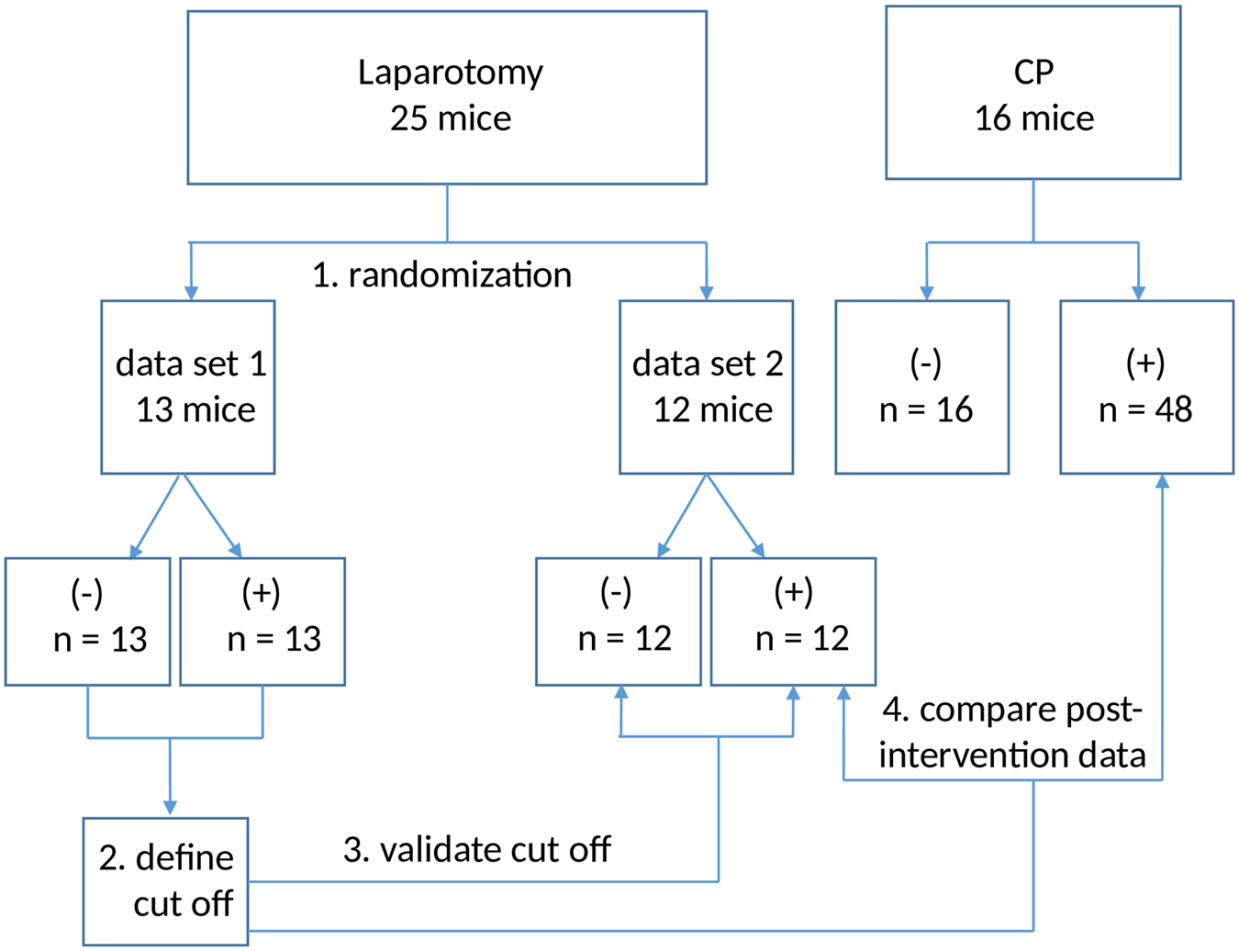
Procedure of distress evaluation. (1.) Data collected from 25 mice (intervention: laparotomy) were separated into two data sets by randomisation. (2.) Data of data set 1 were used to define the cut off, which separates distress observed before (−) from distress observed after intervention (+). (3.) This cut off was validated using data set 2. (4.) This cut off was also used to compare distress after laparotomy to distress during CP.

**Figure 6:**
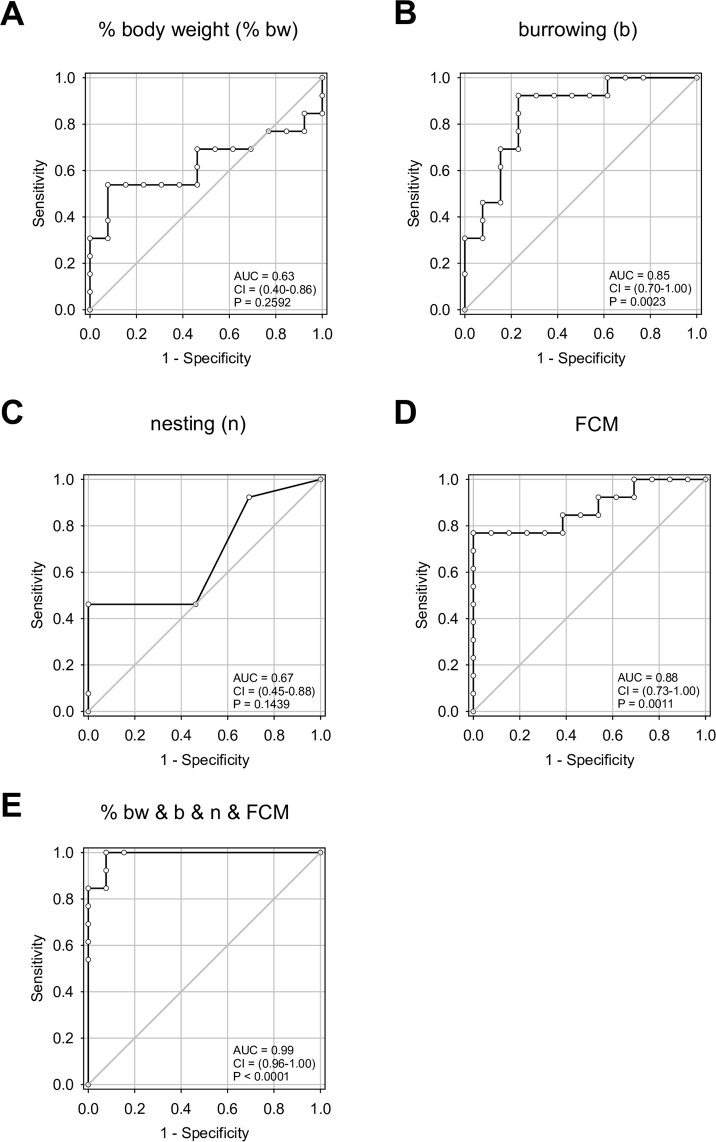
Receiver operating characteristic curve analysis of distress parameters (laparotomy data set 1). (**A**) Percentage of body weight change, (**B**) burrowing activity, (**C**) nesting activity, (**D**) faecal corticosterone metabolites concentration and (**F**) combination of these four parameters after applying binary logistic regression in a multiple model. The area under the curve (AUC) with the respecting confidence interval (CI) and the P value (P) for testing AUC to be 0.5, are indicated. Before induction of laparotomy: n = 13, after induction of laparotomy: n = 13.

After laparotomy the body weight was significantly decreased (Fig. 4A). In addition, the amount of pellets burrowed significantly plummeted after the surgical intervention (Fig. 4B) and the nesting score was significantly reduced after laparotomy (Fig. 4C), while FCM concentration significantly increased (Fig. 4D). These data were separated into two data sets by randomisation (Fig. 5). ROC analysis was performed on data set 1 in order to compare the accuracy of these parameters in defining distress (Fig. 6A–D). The single readout parameters, percentage of body weight change and nesting activity (Fig. 6A,C), had only little discriminatory power to differentiate between animals before and after laparotomy (body weight: 0.63, 0.40–0.86 confidence interval, P = 0.2592; nesting: 0.67, 0.45–0.88 confidence interval, P = 0.1439). Burrowing activity (Fig. 6B) and FCM (Fig. 6D) had a high discriminatory power to differentiate between animals before and after laparotomy. The combination of all four parameters (Fig. 6E) produced the largest area under the curve (AUC = 0.99, 0.96–1.00 confidence interval, P < 0.0001), suggesting that the combination of all readout parameters allows differentiating between animals before and after laparotomy with higher accuracy than any of the single readout parameters (Fig. 6A–E). When determining the best cut off to this multi-parametric analysis, the combination of all readout parameters allows to differentiate between animals before and after laparotomy with higher accuracy (96.2%, 80.4–99.9% confidence interval) than any of the single readout parameters (Supplementary Table S2). In order to check the validity of this cut off, we evaluated if this cut off can differentiate between animals before and after laparotomy using a different data set (data set 2). This cut off had 100% specificity, 100% sensitivity and 100% accuracy when applied to data set 2. When applying this cut off to data taken after laparotomy (data set 2), all data collected after laparotomy were allocated to distress level 2 (Table 1). When applying this cut off to data taken after induction of CP, some data were allocated to distress level 2, whereas some data were allocated to distress level 1, which indicates baseline distress of healthy animals (Table 1). Using Fisher’s exact test, a significant difference in the distress levels distribution between laparotomy and CP was observed. This suggests that during CP two different distress levels can be assessed: A distress level similar to distress observed after laparotomy (distress level 2) and a distress level that compares to baseline distress (distress level 1).

**Table 1:** 2 × 2 contingency table that represents different distribution of distress levels after laparotomy (data set 2, n = 12) and cerulein-induced CP (n = 48) according to combined distress cut off value 0.65, which was defined using the laparotomy data set 1. Fisher’s exact test (*P = 0.012).

	laparotomy (data set 2)	pancreatitis (all data)
distress level 1	0	19
distress level 2	12	29

*Significant different distribution of distress levels after laparotomy and pancreatitis

## Discussion

This study demonstrates that all distress readout parameters (FCM, body weight change and burrowing as well as nesting activity) are significantly changed by both CP and laparotomy. This suggests their broad applicability to assess distress after completely distinct interventions. However, the accuracy of these parameters when distinguishing between healthy mice and mice after an intervention was different between CP and laparotomy. In the animal model of CP body weight change was a more accurate parameter than FCM (accuracy of parameters: body weight > burrowing > nesting > FCM), whereas after laparotomy FCM was the most accurate parameter to measure distress (accuracy of parameters: FCM > burrowing > nesting > body weight). In addition, this study also demonstrates that in both animal models the multi-parametric analysis relying on all four readout parameters had the highest accuracy when predicting distress. Interestingly, these data also suggest that distress does not steadily increase during CP.

Body weight and burrowing as well as nesting activity have been often evaluated when assessing distress of animals^18,19,39^. Reduction of body weight was, for example, reported as a good indicator of distress in acute colitis and after general anaesthesia^10,11,40^. Body weight reduction was also described after induction of CP with cerulein in mice^41^. Moreover, reduced burrowing or nesting activity were observed after laparotomy^42^, induction of hippocampal lesion^15^, isoflurane anaesthesia^18^, or intra-bone marrow transplantation^43^. Analysis of FCM is also a well-established non-invasive method to evaluate distress and incurs less stress on mice than the measurement of corticosterone in blood^17,39,44^. It is less often used in studies than body weight and assessment of animal behaviour, but increased FCM concentration was reported after ketamine and xylazine anaesthesia^11^ as well as after intra-bone marrow transplantation^43^. In agreement with the above cited literature FCM, body weight change, burrowing and nesting activity are significantly changed by CP as well as laparotomy (Figs. 2 and 4). This suggests that these readout parameters are sensitive enough to assess distress after completely distinct interventions, i.e. repetitive cerulein injections and laparotomy. Moreover, we also tested the effect of microRNA-21 inhibitor on these distress parameters. MicroRNA-21 inhibition was described as an effective strategy in many therapeutic areas^45–47^. Thus, we hypothesized that microRNA-21 inhibitor can mitigate animal distress that might be caused by neuropathic pain component of CP^37,38^. This assumption was based on the previously reported contribution of microRNA-21 to neuropathic pain^35,36^. However, application of microRNA-21 inhibitor did not have any significant effect on any of the four distress readout parameters during CP. These negative results might be attributed, at least partly, to the multifactorial nature of pain in CP^33,37^.

Although distress parameters such as FCM, body weight, burrowing as well as nesting activity are often used to measure distress^10,11,18,43^, little is known about the sensitivity, specificity and accuracy of these readout parameters. According to ROC analysis, we found that body weight and burrowing are able to predict distress in CP with a higher accuracy than nesting and FCM. On the other hand, FCM and burrowing were the most accurate single parameters in predicting distress in laparotomy. Thus, although all four readout parameters are broadly applicable to assess distress, the accuracy of these parameters when distinguishing between healthy mice and mice after an intervention was different between CP and laparotomy. This argues for using more than one parameter for evaluating distress of animals. This is especially necessary, when comparing distress caused by distinct interventions. Indeed, our multi-parametric analysis using logistic regression followed by ROC analysis allowed us to estimate the accuracy of multi-parametric analysis using all four parameters. So far, only a few other studies reported multi-parametric analysis with methods such as cluster analysis^10^.

Animal models with long lasting distress such as CP are important for basic or preclinical research on many chronic diseases^48–50^. However, according to the European Union Directive 2010/63/EU, long lasting distress for animals automatically increases the severity of an animal experiment^4^. For example, long-lasting moderate distress has to be upgraded to severe distress^4^. Indeed, we know that long lasting diseases can lead to persistent or even increased distress and pain. This is, for example, true for osteoarthritis or neuropathic pain^51,52^. However, we also know that long lasting stress can lead to habituation or desensitization. One well accepted example is the hypothalamic–pituitary–adrenal axis, where repetitive stress often leads to decreased release of stress hormones such as corticosterone^53,54^. Interestingly, we found that percentage of body weight change, burrowing and nesting activity remained steady between the middle and late phase of CP (Fig. 2A–C). Moreover, FCM concentration was even significantly reduced in the middle phase when compared to the early phase of CP (Fig. 2D). A reduction of the stress hormone corticosterone was also observed in the blood when comparing middle and late phase of disease progression to the early phase of CP (Supplementary Fig. S1). These data suggest that redundant cerulein injections, especially during the late stage of CP, do not lead to hyperalgesia. This is consistent with several publications describing habituation in mice after exposure to repetitive stress^55,56^.

Surprisingly, only few studies assessed distress caused by preclinical *in vivo* research^11,18,43^. Thus, there is an urgent need for detailed characterization of distress in most animal models. In order to judge distress caused by cerulein-induced CP, we used distress caused by laparotomy as a reference, since the European Union Directive 2010/63/EU suggests that the distress level after laparotomy is moderate. When pursuing a multi-parametric analysis using FCM, body weight and burrowing as well as nesting activity of data set 1 before and after laparotomy, a best cut off could be defined that differentiates between ‘baseline distress’ (data taken before laparotomy) and ‘up to moderate distress’ (data taken after laparotomy). Subsequently, this cut off was applied on two different post intervention data sets; laparotomy data set 2 and the CP data set. This comparison demonstrates that after CP, 60% (29 from 48) of all data points can be defined as distress level 2 (up to moderate distress), whereas 40% (19 from 48) of all data points can be defined as distress level 1 (baseline distress). This suggests that at any given time point during CP mice experience sometimes baseline distress and sometimes up to moderate distress. Thus, when just considering one time point, mice often experience less distress during CP than after laparotomy. The following points support such a conclusion. First, none of the mice in both models lost more than 20% of original body weight, a margin which is commonly used to indicate more than moderate, thus severe distress^16^. Second, none of the mice died or had to be euthanized. Consequently, we propose that distress at a certain time point during CP could be defined to be mild to moderate.

However, the European Union Directive 2010/63/EU demands that the continuity of distress has to be taken into account when judging the severity level of an animal model. For example, long term mild distress should be upgraded to an overall moderate distress. Likewise, long term moderate distress should be upgraded to severe distress. Considering these recommendations, the severity level of CP could be defined to be moderate to severe. However, such a classification seems to violate proportionality when one compares CP to animal models and interventions that are suggested to be severe by the European Union Directive 2010/63/EU. For example, this directive suggests to classify toxicity testing where death is the end-point and irradiation with a lethal dose without reconstitution of the immune system as severe^4^. In these cases a very high percentage of animals would suffer until death or euthanasia. In contrast to these scenarios, CP only induced a maximal loss in body weight of 13% at any time point. Moreover, no mouse died or had to be euthanized during CP. This suggests that we need more than four classes (non-recovery, mild, moderate and severe) of defining severity of animal experiments, in order to generate a fair system that guarantees proportionality in the severity scoring of distinct animal models.

In conclusion, this study suggests that ROC analysis is a feasible tool to compare sensitivity, specificity and accuracy of distinct methods to analyse animal distress. Moreover, multi-parametric analysis of distress with these four parameters has the potential to evaluate distress with a high accuracy and allows a comparison between distress caused by completely different interventions such as induction of CP and laparotomy. This multi-parametric analysis revealed that mice during the course of CP often experienced less distress than mice after laparotomy.

## Material and Methods

### Ethical statement

This study was conducted in accordance with the German law for animal protection (TierSchG) and the European Union Directive, 2010/63/EU. The public authority (Landesamt für Landwirtschaft, Lebensmittelsicherheit und Fischerei Mecklenburg-Vorpommern, 7221.3–1–002/17, 7221.3–1–019/15) and the §15 (according to the TierSchG) committee of Mecklenburg-Vorpommern approved all experiments and experimental protocols.

### Animals

C57Bl/6J mice were housed in type III cages at 12 h dark light cycle (light period: 7:00–19:00) and a temperature of 21 ± 2 °C, with a relative humidity of 60 ± 20 %. Food (pellets, V1534.000, 10 mm, ssniff Spezialdiaeten GmbH, Soest, Germany) and water were provided *ad libitum*. Autoclaved bedding (Bedding Espe Max 3–5mm granulate, H 0234–500, Abedd, Vienna, Austria) was used. Enrichment was provided by nesting material (Zoonlab GmbH, Castrop-Rauxel, Germany), a fun tunnel (a 75 × 38 mm paper tunnel, H 0528–151, ssniff Spezialdiaeten GmbH,) and a wooden block (Espe size S, 40 × 16 × 10 mm), H0234.NSG, Abedd).

CP was induced by repetitively injecting male 9–15 weeks old C57Bl/6J mice with cerulein. Cerulein (Bachem H-3220.0005, Bubendorf, Switzerland) dissolved in 0.9% sodium chloride was administered by consecutive intraperitoneal (i.p.) injections (50 μg/kg, three hourly injections/day; three days/week (on day 0, 2, 4, 7, 9, 11, 14, 16, 18, 21, 23, 25, 28 and 30). MicroRNA-21 inhibitor (miRCURY LNA^™^ microRNA-21a-5p inhibitor; cat. #, 339203 YCO0070656, sequence: TCAGTCTGATAAGCT) and its corresponding microRNA-21 control (miRCURY LNA^™^ microRNA-21a-5p control; cat. #, 339203 YCO0070657, sequence: TCAGTATTAGCAGCT) were purchased from Qiagen (Hilden, Germany). They were resuspended in PBS and injected at a dose of 10mg/kg (s.c. to ensure sustained release of the drug) on day 0 and day 14. For pain relief 1250 mg/l metamizol (Ratiopharm, Ulm, Germany) was provided daily (starting at day −1 until day 33) in the drinking water. The mice were euthanized under anaesthesia by cervical dislocation. The pancreas to body weight ratio of each mouse was recorded on day 33 and the tissue was taken for further analysis. Animals (16 mice split into two separate experiments) were allocated in a non-random manner either to a control or a treatment group by matching their burrowing activity during the pre-phase. Both microRNA-21 control and inhibitor were injected in a non-blinded manner.

In order to execute laparotomy, male C57Bl/6J mice were anaesthetized by 1.2–2.5% isoflurane, which provides general anaesthesia as well as an analgesic effect and reduction of stress. The eyes were protected by applying eye ointment. Within one to two minutes carprofen (Pfizer GmbH, Berlin, Germany) at a dosage of 5 mg/kg was injected (s.c.) before surgery. After shaving and disinfection of the skin, the abdominal cavity was opened by transverse incision. Then, 5 μl of the cell suspension (murine cell line 6606PDA, 2.5 × 10^5^ cells in 5 µl matrigel) were injected slowly with a 25 μl syringe into the pancreas (Hamilton Syringe, Reno, Nev., USA). Thereafter, the cavity and the skin were closed by a coated 5–0 vicryl suture (Johnson & Johnson MEDICAL GmbH, Norderstedt, Germany) and a 5–0 prolene suture (Johnson & Johnson MEDICAL GmbH). The purpose of this cell injection was to generate an orthotopic syngeneic pancreatic cancer model to explore drugs for the treatment of cancer (see e.g. Zechner *et al*.^57^ and Zhang *et al*.^58^; the FCM data for this model have been created for the manuscript of Kumstel *et al*. 2019, submitted to Scientific Reports). The data of the laparotomy animal model were randomized to define data set 1 and data set 2 using the random number generator at the official website www.stattrek.com.

### Analysis of animal distress

In the laparotomy model, the percentage of body weight change, burrowing activity, nesting activity, FCM concentrations were measured at two time points: before and after surgical intervention. In the CP model, distress readout parameters were measured, before induction of CP (pre-phase) and throughout the different phases of disease progression; early, middle and late phase. Before each experiment, burrowing and nesting assays were performed two times with mice housed in groups of four or six animals to facilitate that the mice learn this behaviour from each other. Thereafter, all the animals were housed individually and burrowing and nesting activity were assessed three times. Mice which burrowed three times less than 10g or which had an average nesting score of less than 2.5 during the pre-phase were excluded from further analysis.

The body weight was measured two times before and one day after laparotomy as well as before induction of CP (pre-phase) and throughout the different phases of disease progression; early (day 3), middle (day 17) and late (day 31). Thus, in all experiments the body weight was determined 24 hours after induction of distress.

The burrowing activity was measured before and on the day of laparotomy as well as before induction of CP (pre-phase) and throughout the different phases of disease progression; early (day 2), middle (day 16) and late (day 30). In order to quantify burrowing activity, a burrowing tube (15 cm length × 6.5 cm diameter) was filled with 200 ± 1 g food pellets (ssniff Spezialdiaeten GmbH) and was put in the cage two and a half to three hours before the dark phase. After two hours, the weight of the food pellets [g] left in the tube was measured and deducted from 200g.

Nesting activity was measured before and on the day of laparotomy as well as before induction of CP (pre-phase) and throughout the different phases of disease progression; early (day 2), middle (day 16) and late (day 30). In order to analyse nest building behaviour, a cotton nestlet (5 cm square of pressed cotton batting, Zoonlab GmbH, Castrop-Rauxel, Germany) was placed in the left back corner of the cage 30 to 60 minutes before the dark phase. The nests were scored at the end of the dark phase ±2 hour, by using a scoring system developed by Deacon^15^. However, a 6^th^ score point was added to this scoring system. The score 6 defines a perfect nest: The nest looks like a crater and more than 90% of the circumference of the nest wall is higher than the body height of the coiled up mouse.

FCM was assessed in faeces (which was left in the cages within 24 hours) before and on the day of laparotomy as well as before induction of CP (pre-phase) and throughout the different phases of disease progression; early (faeces collected from day 2 to day 3), middle (day 16–17) and late (day 30–31). In the laparotomy model, faeces was collected before and during 24 hours immediately after the surgical intervention. The faeces (200–400 mg) was dried for 4 hours at 65 °C and kept at −20 °C until further processing. Thereafter, 50 mg of dried faeces were extracted with 1 ml 80% methanol for further analysis by 5α-pregnane-3β,11β,21-triol-20-one enzyme immune assay^17,59^.

Corticosterone concentration was evaluated exclusively in the blood plasma of animals other than those subjected to distress analysis by non-invasive methods (four additional animals). Thus, the analysis of distress by non-invasive methods was not influenced by blood collection. The blood samples were taken before induction of CP without any intervention (pre-phase) and throughout the different phases of disease progression; early (day 2), middle (day 16) and late (day 30) between 11.00 and 15.30 h to refrain from the circadian increase of plasma corticosterone after 16.00 h^60,61^. Blood was collected 30 minutes after the last cerulein injection by retro-orbital puncture. This time interval is important because the plasma corticosterone concentration is highest 30 minutes after cerulein injection^62^. Mice were placed into a chamber filled with 5% isoflurane and 100–150µl blood was taken within three minutes of the onset of anaesthesia. The rationale behind using retro-orbital puncture is that this method yields a rather large volume of 100 µl of blood in a short time interval of 10 sec without the need of subsequent manual haemostasis. Blood sampling within three minutes is mandatory in order to exclude that the sampling procedure increases the corticosterone concentration^63,64^. Retro-orbital puncture is more than seven times faster than saphenous vein puncture and more than 15 times faster than tail vein puncture when collecting the same volume of blood^65^. Blood sampling was performed by a well experienced researcher. Blood samples were centrifuged at 1200 × g for 10 minutes and stored at −20 °C. Plasma corticosterone concentration was measured using an ELISA-Kit (DEV 9922, Demeditec Diagnostics GmbH, Erfurt, Germany) according to the manufacturer‘s instructions.

### Lipase activity and immunohistochemistry

Lipase activity was detected in blood plasma by colorimeter (Cobas C111, Roche diagnostics GmbH, Mannheim, Germany) using a kit (LIPC REF # 05401704, Roche diagnostics GmbH, Mannheim, Germany) according to the manufacturer’s instructions. Immunohistochemistry was performed using a rabbit polyclonal anti-collagen I primary antibody (ab34710, Abcam, Cambridge, UK) at 1:200 dilution and a goat anti-rabbit AP-conjugated secondary antibody at a 1:100 dilution (D0487, Dako, Hamburg, Germany). The pre-treated slides were incubated with permanent red solution (K0640, Dako) prepared according to the manufacturer’s instructions. Image J 1.52a software (National Institutes of Health, USA) was used to quantify collagen I in an automated manner.

### Graphs and statistical analysis

All box plots represent the 10th and 90th percentile as whiskers and all line graphs show mean and standard deviation. They were graphed using SigmaPlot 12.0 (SYSTAT Software Inc., San Jose, USA). All biostatistical analysis was done using SigmaPlot 12.0 (SYSTAT Software Inc., San Jose, USA), except for the t-test with Welch's correction, which was performed using GraphPad prism 6.0 (GraphPad Software, San Diego, USA). Significances of differences were evaluated using One Way Repeated Measures ANOVA, Two Way Repeated Measures ANOVA, Student t-test, t-test with Welch’s correction, Mann-Whitney rank sum test, paired t-test or Wilcoxon signed-rank test (see figure legends) according to data characteristics (the decision was based on the fact, if the data were paired and if they passed the Shapiro-Wilk normality test and the Levene median test for equal variance). Differences with p ≤ 0.05 were considered to be significant.

In order to compare the validity of parameters for defining distress, ROC analysis was performed. The data of all animals before and after intervention (either induction of CP or laparotomy) were used for distress prediction and the area under the curve, the 95% confidence intervals, the p-value and the best cut off (based on the highest calculated Youden’s index) were calculated for each parameter. ROC curves present the accuracy of a diagnostic test (an area under the curve of 1.0 means that the parameter is perfect to discriminate between the animals before and after the intervention, whereas a value of 0.5 indicates no discriminative power for this parameter). To analyze the efficacy of the combination of all four parameters, the data sets were combined by binary logistic regression in a multiple model and the ROC curves were calculated afterwards. The best cut off of this multi-parametric distress analysis was calculated using data set 1 of the laparotomy experiment. This cut off was applied to laparotomy data set 2 for verification. The identical cut off was then applied to classify distress after induction of laparotomy or induction of CP in form of a 2 × 2 contingency table. Subsequently, the Fisher‘s exact test was pursued to determine significances in the distribution of two distinct distress levels after laparotomy and cerulein-induced CP.

## Supplementary information

Supplementary information

## Data Availability

The datasets used and/or analyzed during the current study are available from the corresponding authors on reasonable request.
