# Supplementary material for: A novel multi-parametric analysis of non-invasive methods to assess animal distress during chronic pancreatitis
Source: Sci Rep. 2019 Oct 1;9:14084. doi: 10.1038/s41598-019-50682-3 (PMC6773730; doi:10.1038/s41598-019-50682-3)
Supplement: Supplementary file 1 — Supplementary information [file 41598_2019_50682_MOESM1_ESM.pdf]

A novel multi-parametric analysis of non-invasive methods to assess animal distress during chronic pancreatitis

Ahmed Abdelrahman[1*], Simone Kumstel[1], Xianbin Zhang[1], Marie Liebig[1], Edgar Heinz Uwe Wendt[1], Johanna Eichberg[1], Rupert Palme[2], Thomas Thum[3], Brigitte Vollmar[1] and Dietmar Zechner[1*]

[1]Rudolf-Zenker-Institute of Experimental Surgery, Rostock University Medical Center, Rostock, Germany
[2]Unit of Physiology, Pathophysiology and Experimental Endocrinology, Department of Biomedical Sciences, University of Veterinary Medicine Vienna, Vienna, Austria
[3]Institute of Molecular and Translational Therapeutic Strategies, Hannover Medical School, Hannover, Germany

*Corresponding authors:
Ahmed Abdelrahman Institute for Experimental Surgery, Rostock University Medical Center, Schillingallee 69a, 18057 Rostock, Germany. Tel: +493814942506, Fax: +493814942502,
E-mail: Ahmed.Abdelrahman@med.uni-rostock.de

PD Dr. rer. nat. Dietmar Zechner, Institute for Experimental Surgery, Rostock University Medical Center, Schillingallee 69a, 18057 Rostock, Germany. Tel: +493814942512, Fax: +493814942502,
E-mail: dietmar.zechner@uni-rostock.de

Supplementary Fig. S1

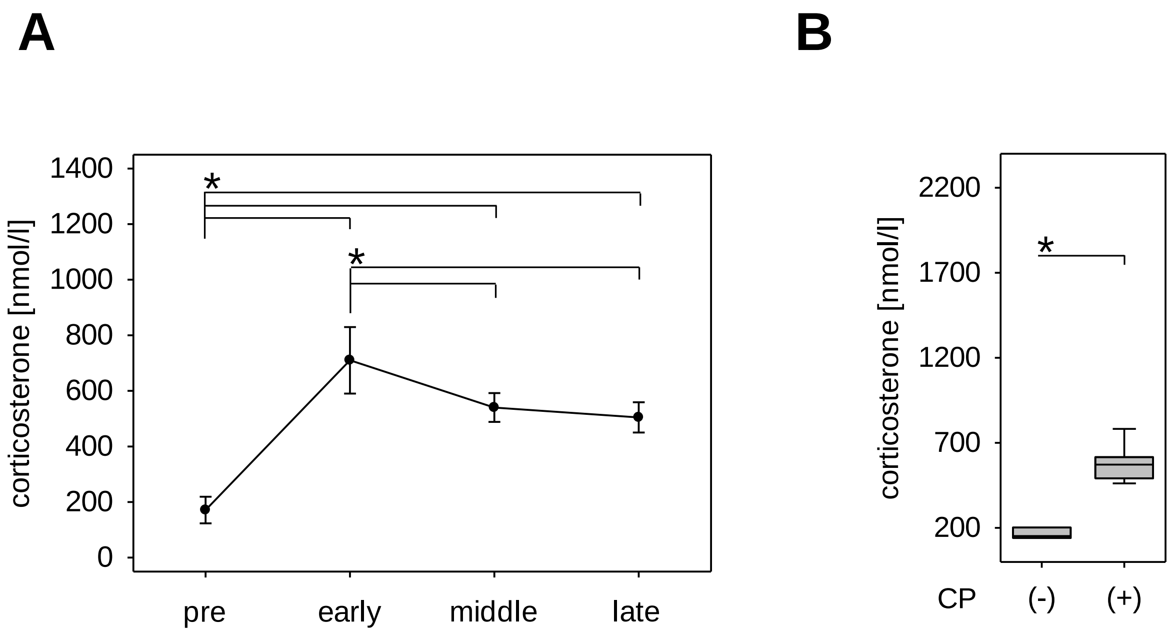

Supplementary Figure 1. Determination of corticosterone concentration in blood plasma. A) Corticosterone concentration before (pre) and during the early, middle and late phases of CP; One Way Repeated Measures Analysis of Variance, followed by pairwise comparison with Holm-Sidak-Method (*P ≤ 0.003), n = 4. B) Comparing corticosterone concentration before (- CP, n = 4) and during early, middle and late phase of disease progression (+ CP, n = 12); Mann-Whitney Rank Sum Test (*P = 0.004).

Supplementary Table S1

Supplementary table 1. Comparison of the best cut off values. The best cut off values and the respecting specificity, sensitivity as well as accuracy of this cut off in the cerulein-induced CP animal model; before induction of CP: n = 16, after induction of CP. n = 48. CI = confidence interval.

Read out parameter	best cut off	specificity (CI)	sensitivity (CI)	accuracy (CI)
% body weight change (% bw)	-1.32	87.5 % (61.7%-98.5%)	87.5% (74.8%-95.3%)	87.5% (76.9%-94.5%)
burrowing (b)	72.00	87.5 % (61.7%-98.5%)	70.8% (55.9%-83.1%)	75.0% (62.6%-85.0%)
nesting (n)	4.50	87.5 % (61.7%-98.5%)	70.8% (55.9%-83.1%)	75.0% (62.6%-85.0%)
FCM	698.50	93.8 % (69.8%-99.8%)	52.1% (37.2%-66.7%)	62.5% (49.5%-74.3%)
% bw & b & n & FCM	0.43	93.8 % (69.8%-99.8%)	97.9% (88.9%-100.0%)	96.9% (89.2%- 99.6%)

Supplementary Table S2.

Supplementary table 2. Comparison of the best cut off values. The best cut off values and the respecting specificity, sensitivity as well as accuracy of this cut off using the laparotomy animal model; data set 1. Before induction of laparotomy: n = 13, after induction of laparotomy: n = 13. CI = confidence interval.

Read out parameter	best cut off	specificity (CI)	sensitivity (CI)	accuracy (CI)
% body weight change (% bw)	-2.73	92.3 % (64.0%-99.8%)	53.9% (25.1%-80.8%)	73.1% (52.2%-88.4%)
burrowing (b)	140.00	76.9 % (46.2%-95.0%)	92.3% (64.0%-99.8%)	84.6% (65.1%-95.6%)
nesting (n)	2.50	100.0 % (75.3%-100.0%)	46.2% (19.2%-74.9%)	73.1% (52.2%-88.4%)
FCM	989.90	100.0 % (75.3%-100.0%)	76.9% (46.2%-95.0%)	88.5% (69.9%-97.6%)
% bw & b & n & FCM	0.65	92.3 % (64.0%-99.8%)	100.0% (75.3%-100.0%)	96.2% (80.4%-99.9%)
